# From missed diagnosis to lifelong survivorship: a precision oncology roadmap for equity in pediatric and AYA cancer care

**DOI:** 10.3389/fonc.2026.1982346

**Published:** 2026-10-13

**Authors:** Emmanuel Ifeanyi Obeagu

**Affiliations:** 1 Department of Biomedical and Laboratory Science, Africa University, Mutare, Zimbabwe; 2 Department of Molecular Medicine and Haematology, University of the Witwaterarand, Johannesburg, South Africa; 3 Department of Hematology and Immunohematology, Bahir Dar University, Bahir Dar, Ethiopia

**Keywords:** adolescents and young adults, cancer health equity, cancer survivorship, pediatric oncology, precision oncology

## Abstract

Cancer in children, adolescents, and young adults (AYA) remains a global health challenge, with disparities in survival and quality of life between high-resource and low- and middle-income countries (LMICs). These disparities arise across the cancer continuum, including delayed recognition, inadequate diagnostic capacity, fragmented referral pathways, treatment abandonment, limited supportive care, unequal access to molecular diagnostics, and insufficient survivorship services. Precision oncology provides an opportunity to address these challenges, but its equity potential extends beyond genomic sequencing and targeted therapies. This narrative review examines an integrated precision oncology continuum encompassing early recognition, diagnostic and pathological accuracy, molecular characterization, risk stratification, precision treatment, response monitoring, supportive care, and lifelong survivorship. Evidence from LMIC settings is used to illustrate diagnostic delay, referral barriers, treatment abandonment, health-system limitations, and outcome disparities without attempting an exhaustive country comparison. The review proposes a tiered, equity-centered framework in which foundational cancer services are strengthened alongside progressively accessible molecular and digital technologies. Regional referral networks, patient navigation, tele-oncology, pathology and laboratory networks, targeted molecular testing, multidisciplinary care, survivorship planning, and longitudinal cancer registries are emphasized as practical components. For AYA patients, fertility, reproductive health, mental health, education, employment, and transition between pediatric and adult services are incorporated into precision care. Ultimately, equitable precision oncology should connect biological precision with health-system precision, ensuring that every young person can receive timely diagnosis, appropriate treatment, continuous monitoring, and lifelong survivorship support.

## Introduction

Cancer in children, adolescents, and young adults (AYA) remains an important global health challenge, with outcomes shaped by a complex interaction between tumor biology, timely diagnosis, treatment availability, health-system capacity, socioeconomic conditions, and continuity of care. Although substantial improvements in survival have been achieved for many childhood and AYA cancers in high-resource settings, major disparities persist across and within countries. These disparities are particularly pronounced in low- and middle-income countries (LMICs), where patients may encounter delayed recognition, limited diagnostic capacity, advanced disease at presentation, shortages of essential medicines and supportive-care resources, treatment abandonment, and inadequate long-term follow-up. Consequently, survival disparities cannot be explained solely by differences in tumor biology; they also reflect inequities throughout the cancer-care pathway (1, 2). The AYA population presents additional challenges because it occupies a transitional space between pediatric and adult oncology. AYA patients have distinctive distributions of malignancies, biological characteristics, developmental needs, treatment priorities, and psychosocial circumstances. Fragmentation between pediatric and adult services can compromise continuity of care, while concerns related to fertility, education, employment, mental health, sexuality, family relationships, and financial independence may remain insufficiently integrated into cancer management. For survivors, the consequences of treatment may persist for decades, creating a need for structured surveillance and individualized survivorship care (3–5).

Precision oncology offers an opportunity to address these challenges by moving beyond a uniform approach to cancer management toward care informed by the clinical, pathological, molecular, and individual characteristics of each patient. In this review, precision oncology is conceptualized as an integrated continuum encompassing clinical precision, diagnostic and pathological precision, molecular precision, risk stratification, therapeutic precision, response monitoring, and survivorship precision. This broader conceptualization is particularly relevant to pediatric and AYA cancer because precision begins with recognizing cancer promptly and establishing an accurate diagnosis; genomic testing and targeted therapy cannot compensate for failures occurring earlier in the care pathway (6–8). This narrative review examines the major barriers and opportunities across this continuum and proposes an equity-centered precision oncology roadmap for pediatric and AYA cancer care, with particular attention to LMICs. The review moves from missed or delayed diagnosis to integrated molecular characterization, risk-adapted treatment, response monitoring, survivorship, digital innovation, and learning health systems. The central premise is that the ultimate measure of precision oncology is not the sophistication of the technology deployed, but whether it enables every child, adolescent, and young adult with cancer to receive an accurate diagnosis, appropriate treatment, continuous care, and the opportunity for a healthy and productive life.

## Methods

This narrative review was developed to synthesize contemporary evidence on precision oncology, diagnostic equity, treatment disparities, survivorship, and health-system approaches relevant to pediatric and adolescent and young adult (AYA) cancer care, with particular emphasis on low- and middle-income countries (LMICs). Literature was identified through searches of major biomedical and multidisciplinary databases, including PubMed/MEDLINE, Scopus and Web of Science. Search terms and combinations included *pediatric cancer*, *childhood cancer*, *adolescent and young adult cancer*, *AYA oncology*, *precision oncology*, *precision medicine*, *diagnostic delay*, *cancer inequity*, *health disparities*, *molecular diagnostics*, *genomics*, *treatment abandonment*, *survivorship*, *LMICs*, *low-resource settings*, and related terms. Reference lists of relevant articles, narrative and systematic reviews, consensus documents, and authoritative reports were also manually examined to identify additional publications of clinical or conceptual relevance. Priority was given to peer-reviewed studies, systematic and narrative reviews, international guidelines, consensus statements, major cancer reports, and authoritative publications addressing pediatric and AYA oncology. Both foundational literature and recent evidence were considered where necessary to provide historical context and describe developments in precision diagnostics, molecular classification, targeted treatment, digital oncology, and survivorship. English-language publications were primarily considered, reflecting the language of the accessible literature and the scope of the review. No formal date restriction was imposed when older publications provided important foundational evidence; however, emphasis was placed on contemporary evidence where current clinical practice or technological developments were discussed.

As a narrative review, the objective was to provide an integrative and clinically oriented synthesis rather than a systematic estimate of the literature or pooled effect sizes. Articles were selected based on their relevance to the major themes of the review, including early cancer recognition and diagnostic equity, pathological and molecular characterization, risk stratification, precision treatment, treatment access and abandonment, supportive care, artificial intelligence and digital health, survivorship, and implementation of precision oncology in LMICs. Particular attention was given to literature illustrating differences in diagnostic pathways, treatment capacity, referral systems, and survivorship resources across resource settings. Selected country-specific examples were incorporated where they provided useful empirical context, without assuming that findings from individual countries were representative of all LMICs. The evidence was synthesized thematically across the cancer-care continuum, from recognition and diagnosis to treatment, response monitoring, survivorship, and longitudinal health-system learning. The proposed precision oncology framework represents the authors’ conceptual synthesis of the evidence rather than a formally validated clinical algorithm. Emphasis was placed on distinguishing established evidence from proposed strategies and on identifying areas where evidence remains limited, heterogeneous, or context dependent. No quantitative meta-analysis or formal risk-of-bias assessment was undertaken because of the narrative and conceptual nature of the review.

### The pediatric and AYA cancer equity problem

Cancer in children, adolescents, and young adults (AYA) represents a major global health challenge in which outcomes are shaped not only by tumor biology but also by the timeliness, quality, affordability, and continuity of care. Although many childhood and AYA cancers are potentially curable, survival and long-term quality of life remain substantially unequal across regions. These disparities reflect differences in early recognition, diagnostic accuracy, referral systems, treatment availability, supportive care, socioeconomic conditions, and survivorship services. Consequently, precision oncology in this population must address both biological heterogeneity and the health-system conditions that determine whether precision can be delivered in practice (9). The first equity gap occurs at cancer recognition and referral. Early manifestations of childhood and AYA cancers may be nonspecific and can overlap with common infectious, nutritional, inflammatory, or benign conditions. In settings where primary healthcare providers have limited oncology training, diagnostic pathways may involve repeated consultations before referral to an appropriate specialist. Geographic distance, transportation costs, fragmented referral systems, and concentration of oncology services in tertiary institutions can further prolong the interval between symptom onset and definitive assessment. Diagnostic delay may result in advanced disease, greater treatment intensity, complications, and reduced opportunities for curative intervention (10).

A second major challenge is diagnostic precision. Accurate cancer classification increasingly depends on coordinated clinical assessment, pathology, immunophenotyping, cytogenetics, molecular diagnostics, and appropriate imaging. However, access to these capabilities is uneven, particularly in resource-constrained settings. Limited pathology infrastructure, shortages of trained personnel, inadequate specimen transportation, reagent interruptions, and restricted access to molecular testing can lead to diagnostic uncertainty or treatment based on incomplete biological information. Importantly, precision oncology should therefore not be equated with advanced genomic sequencing alone; reliable foundational pathology and laboratory systems are prerequisites for meaningful molecular precision (11). Treatment inequity further widens differences in outcomes. Even when a diagnosis is established, patients may encounter shortages of essential medicines, chemotherapy agents, blood products, radiotherapy, surgery, intensive care, or specialist expertise. High treatment costs and the need for prolonged travel can impose substantial financial and social burdens on families. Treatment abandonment should consequently be understood within the broader context of health-system and socioeconomic barriers rather than solely as a failure of patient or caregiver adherence. Patient navigation, financial and social support, decentralized services, treatment coordination, and effective communication can therefore become important components of precision care (12).

Supportive care represents another critical dimension of equity. Infection prevention and management, transfusion support, nutritional care, pain control, management of treatment toxicity, psychosocial services, and palliative care can determine whether patients tolerate and complete potentially curative treatment. A treatment regimen that is biologically appropriate but cannot be safely delivered because supportive-care infrastructure is inadequate does not constitute effective precision oncology. Thus, supportive-care precision should be integrated with diagnostic and therapeutic precision (9). AYA patients face additional challenges because they occupy a transitional space between pediatric and adult oncology. Their cancers may differ biologically and clinically from those commonly encountered in younger children, while their developmental needs introduce additional considerations involving fertility, reproductive health, mental health, education, employment, sexuality, financial independence, and family relationships. Fragmentation between pediatric and adult oncology services can create gaps in referral, treatment continuity, psychosocial support, and survivorship planning. Structured AYA pathways and coordinated transition models are therefore essential components of equitable cancer care (10). The pediatric and AYA cancer equity problem is therefore best understood as a continuum-of-care problem rather than a single diagnostic or therapeutic deficit. An equitable precision oncology model must connect recognition, referral, accurate diagnosis, molecular characterization, risk stratification, treatment, supportive care, response monitoring, survivorship, and lifelong follow-up. In LMICs, this approach requires strengthening foundational cancer services while progressively integrating molecular, digital, and data-driven technologies according to clinical need, feasibility, and sustainability (**Table 1**).

**Table 1:** The pediatric and AYA cancer equity problem across the cancer-care continuum.

Cancer-care domain	Principal equity challenge	Consequence for children and AYA patients	Precision oncology response
Early recognition	Limited cancer awareness and nonspecific presentations	Delayed referral and more advanced disease	Clinical education, referral triggers, decision-support tools
Diagnosis	Limited pathology, imaging, laboratory, and molecular capacity	Misdiagnosis, diagnostic delay, and uncertain classification	Integrated pathology, immunophenotyping, cytogenetics, and molecular diagnostics
Referral pathways	Fragmented or geographically concentrated specialist services	Delayed access to definitive oncology care	Regional referral networks, navigation, tele-oncology
Risk stratification	Limited access to prognostic and molecular testing	Uniform treatment despite biological heterogeneity	Clinical, pathological, molecular, and response-based risk assessment
Treatment access	Shortages or high costs of medicines, technology, and specialist services	Treatment delays, modification, or undertreatment	Context-adapted precision treatment and strengthened essential-care systems
Treatment abandonment	Transportation, financial, social, and communication barriers	Interrupted therapy and poorer outcomes	Patient navigation, social support, decentralized care, and adherence monitoring
Supportive care	Limited transfusion, infection, nutritional, pain, and psychosocial services	Treatment-related morbidity and mortality	Individualized supportive-care pathways
Response monitoring	Limited access to advanced imaging and measurable residual disease testing	Delayed recognition of treatment failure or relapse	Risk-adapted clinical, laboratory, imaging, and molecular monitoring
AYA-specific care	Fragmented pediatric–adult services and limited age-appropriate support	Gaps in continuity, fertility, education, mental health, and reproductive care	Integrated AYA pathways and structured transition programs
Survivorship	Limited long-term surveillance and late-effect services	Preventable morbidity and reduced quality of life	Individualized survivorship plans and lifelong follow-up
Data and research	Underrepresentation in registries, genomic datasets, and clinical research	Limited evidence for locally relevant precision care	Regional registries, interoperable data systems, and inclusive research
Health-system capacity	Unequal distribution of specialized infrastructure and expertise	Persistent geographic and socioeconomic disparities	Tiered precision oncology networks linking local and specialist services

### Precision oncology: beyond genomics

Precision oncology should extend beyond genomic profiling to encompass the biological, clinical, social, and health-system factors that determine whether a patient receives the right care at the right time. Although molecular characterization can refine diagnosis, risk stratification, therapeutic selection, and disease monitoring, genomic information alone cannot overcome delayed diagnosis, fragmented referral, treatment toxicity, medicine shortages, financial barriers, or treatment abandonment. A broader precision framework therefore integrates diagnostic precision, molecular precision, therapeutic precision, supportive-care precision, adherence precision, and survivorship precision. This approach aligns treatment decisions with tumor biology while simultaneously accounting for age, comorbidities, nutritional status, toxicity risk, psychosocial circumstances, geographic access, and health-system capacity. In pediatric and AYA oncology, such whole-pathway precision is particularly important because successful cancer care extends from early recognition and accurate diagnosis through treatment completion, late-effect surveillance, psychosocial recovery, and transition to adult services. Thus, precision oncology should be understood not merely as matching a molecular alteration to a targeted therapy, but as continuously matching the patient, disease, treatment, resources, and long-term needs across the cancer continuum (13, 14).

### Earlier cancer recognition and diagnostic equity

Earlier cancer recognition is the first step toward equitable precision oncology because accurate treatment cannot compensate for delayed diagnosis. In pediatric and AYA populations, nonspecific symptoms may initially resemble common infections, nutritional disorders, inflammatory conditions, or benign disease, contributing to repeated healthcare visits and delayed referral (15–17). Diagnostic inequity is further amplified by limited access to pathology, imaging, immunophenotyping, molecular testing, and specialist oncology services, particularly in resource-constrained settings (18, 19). Improving recognition therefore requires strengthened clinical awareness, standardized referral criteria, rapid-access oncology pathways, and decision-support tools that identify persistent or unexplained warning signs (20–23). Diagnostic networks should combine essential laboratory and imaging services with tiered access to specialized pathology and molecular platforms, supported where necessary by telepathology and regional referral laboratories. Importantly, equity requires minimizing disparities in diagnostic timeliness across geographic, socioeconomic, and healthcare-access groups (24–27). Monitoring the interval from first presentation to referral, definitive diagnosis, and treatment initiation can identify critical points of system failure (28, 29). Earlier recognition should therefore be regarded not simply as an awareness intervention, but as a precision health-system strategy that improves diagnostic accuracy, reduces preventable delays, and increases the opportunity for curative treatment across diverse pediatric and AYA populations (3, 30).

### Precision diagnostics: the foundation of precision treatment

Precision treatment depends fundamentally on accurate, timely, and clinically integrated diagnosis. In pediatric and AYA oncology, diagnostic precision requires the coordinated use of clinical assessment, hematological and biochemical investigations, imaging, histopathology, immunophenotyping, cytogenetics, and molecular testing according to disease-specific needs. Molecular characterization can refine tumor classification, identify prognostic and predictive biomarkers, support risk stratification, guide targeted therapy, and enable measurable residual disease monitoring. However, advanced genomic testing should complement rather than replace essential diagnostic infrastructure. A tiered diagnostic model that combines reliable basic pathology with regional access to specialized molecular platforms can expand precision care while remaining feasible in resource-constrained settings. Digital pathology, telepathology, standardized specimen pathways, external quality assurance, and multidisciplinary interpretation can further reduce diagnostic disparities where specialist expertise is limited. Equally important is ensuring that diagnostic results are available rapidly enough to influence clinical decisions. Thus, precision diagnostics should be evaluated not only by technological sophistication, but also by accuracy, timeliness, clinical utility, accessibility, and equity. Building this diagnostic foundation transforms molecular information into actionable clinical knowledge and enables children and AYAs, regardless of geographic or socioeconomic circumstances, to receive appropriately risk-adapted and biologically informed cancer treatment (**Table 2**) (31).

**Table 2:** Precision diagnostics: the foundation of precision treatment.

Diagnostic level	Key tools	Precision value
Clinical	History, examination, warning signs	Early cancer recognition
Laboratory	CBC, blood film, biochemistry	Initial detection and characterization
Pathology	Histology, cytology, immunohistochemistry	Accurate tumor classification
Hematology	Bone marrow, flow cytometry	Lineage and disease classification
Molecular	PCR, FISH, targeted NGS	Genetic diagnosis and risk stratification
Response monitoring	MRD, molecular assays, imaging	Early detection of treatment response/relapse
Integrated diagnosis	Clinical + pathological + molecular data	Individualized treatment selection

### Artificial intelligence and digital precision oncology

AI could influence pediatric and AYA oncology across the entire care pathway. Machine-learning systems may assist with symptom recognition, imaging interpretation, digital pathology, molecular classification, risk prediction, treatment-response assessment, and survivorship monitoring. AI-enabled imaging could potentially help identify subtle radiological patterns that might otherwise be overlooked. Digital pathology could support remote expert review and reduce geographical disparities in specialist interpretation. Natural-language processing could extract clinically relevant information from medical records and identify patients requiring survivorship surveillance. However, AI also presents substantial equity risks. Algorithms trained predominantly on data from high-income populations may perform poorly in underrepresented populations. Differences in disease prevalence, genetic ancestry, laboratory methods, imaging equipment, nutrition, comorbidities, and treatment pathways can influence algorithm performance. Therefore, algorithmic precision must be accompanied by population precision. AI systems should undergo external validation across diverse populations, transparent performance assessment, bias monitoring, human oversight, and appropriate governance. In resource-constrained environments, AI should solve high-value problems rather than simply introduce sophisticated technology (32).

### Risk stratification: moving from uniform treatment to adaptive care

Risk stratification enables pediatric and AYA oncology to move beyond uniform treatment toward individualized, response-adapted care (33, 34). By integrating disease subtype, clinical characteristics, tumor burden, cytogenetic and molecular features, treatment response, measurable residual disease, and relevant patient factors, clinicians can distinguish patients with different risks of treatment failure, relapse, and toxicity. This approach supports calibrated treatment intensity—escalating therapy when disease risk warrants it while minimizing unnecessary exposure in patients likely to achieve durable remission with less intensive approaches (35–37). Importantly, risk assessment should be dynamic rather than determined solely at diagnosis. Sequential evaluation of treatment response and measurable residual disease can provide opportunities to modify therapy as disease biology and treatment sensitivity become clearer (38, 39). In resource-constrained settings, risk stratification should prioritize validated, clinically actionable markers while progressively incorporating advanced molecular technologies as diagnostic capacity expands. Such adaptive care requires standardized protocols, reliable laboratory systems, multidisciplinary interpretation, and timely communication of results (14, 40). Precision risk stratification seeks to align treatment intensity with disease risk and individual vulnerability, improving therapeutic effectiveness while reducing avoidable toxicity and inequitable treatment outcomes (**Table 3**) (13).

**Table 3:** Riskstratification: moving from uniform treatment to adaptive care.

Risk factor	Assessment	Clinical application
Patient factors	Age, comorbidities, performance status	Treatment intensity adjustment
Disease burden	Stage, tumor size, WBC count	Baseline risk classification
Tumor biology	Cytogenetic and molecular abnormalities	Prognosis and targeted therapy
Treatment response	MRD, imaging, clinical response	Dynamic risk reassessment
Treatment toxicity	Organ function, pharmacogenomic factors	Dose and regimen modification
Relapse risk	Molecular profile + response patterns	Intensified or alternative therapy
Integrated risk	Clinical + biological + response data	Individualized adaptive treatment

### Precision therapeutics and the equity challenge

Precision therapeutics has emerged as one of the most transformative developments in contemporary oncology, promising to move cancer treatment away from a predominantly disease-centered model toward therapy tailored to the biological characteristics of an individual patient’s malignancy. In pediatric and AYA oncology, this transformation is particularly compelling because cancers arising during these developmental stages are frequently driven by distinctive molecular, cellular, and developmental mechanisms. Targeted therapies, immunotherapies, antibody-drug conjugates, bispecific antibodies, and cellular therapies have created new possibilities for patients with selected malignancies, particularly those with relapsed or refractory disease. Yet the remarkable promise of precision therapeutics is accompanied by a fundamental equity challenge: the patients most likely to benefit from therapeutic innovation are not necessarily the patients who can access it (41). Precision therapeutics is often presented as a straightforward sequence: identify a molecular alteration, match it to an effective drug, and achieve better outcomes. In practice, the pathway is considerably more complex. A clinically actionable molecular alteration is useful only when it can be accurately detected, interpreted, linked to an evidence-based intervention, and translated into a treatment that the patient can safely receive. The therapeutic pathway therefore extends from diagnosis to molecular characterization, treatment selection, drug availability, monitoring, toxicity management, treatment completion, and long-term follow-up. Failure at any point can transform a theoretically precise therapy into an inaccessible or ineffective intervention (42).

This distinction is particularly important in pediatric and AYA oncology because children are not simply small adults, and AYA patients are not simply older children. Drug metabolism, organ maturation, immune development, growth, endocrine function, fertility, neurocognitive development, and psychosocial circumstances can substantially influence the therapeutic balance between efficacy and toxicity. A treatment that produces tumor regression but causes severe lifelong organ damage may not represent the optimal therapeutic choice for a child expected to live for many decades. Precision therapeutics must therefore consider not only which treatment can kill the cancer, but also which treatment can control the disease while preserving the patient’s future health (14). The first prerequisite for precision therapeutics is accurate biological characterization. Molecular alterations can identify therapeutic vulnerabilities, but their clinical significance varies considerably. Some genomic abnormalities are directly actionable, whereas others are prognostic, biologically interesting, or of uncertain significance. The presence of a mutation should therefore not automatically trigger treatment with a targeted agent. Molecular findings must be interpreted in relation to disease context, evidence strength, treatment availability, previous therapy, age-specific data, and the patient’s clinical condition (41). In pediatric cancer, this distinction is especially relevant because many molecularly targeted therapies have been studied more extensively in adults than in children. Pediatric tumors may contain actionable alterations, but the evidence supporting specific targeted interventions can be limited by small patient populations, rare disease subtypes, limited clinical trials, and challenges in conducting adequately powered studies. The translation of adult evidence to pediatric patients therefore requires careful consideration of pharmacokinetics, pharmacodynamics, developmental biology, toxicity, and long-term outcomes (43).

### Treatment abandonment: the hidden enemy of cure

Treatment abandonment remains one of the most underrecognized barriers to successful pediatric and AYA cancer care, particularly in low- and middle-income countries (LMICs). It occurs when a patient starts cancer treatment but subsequently fails to complete the recommended therapeutic pathway or is lost to follow-up. In diseases where cure depends on prolonged, protocol-driven therapy, abandonment can transform a potentially curable malignancy into a preventable cause of death. Thus, improving survival requires not only discovering better treatments but ensuring that patients can start, continue, and complete the treatment they have already been offered (44). Abandonment is rarely a simple decision by the patient or family. It is often the endpoint of multiple interacting pressures, including poverty, transportation costs, long distances to oncology centers, accommodation difficulties, food insecurity, loss of caregiver income, treatment toxicity, medication shortages, inadequate communication, fear, stigma, and limited understanding of the disease. Families may be willing to continue treatment but lack the resources required to remain within a highly centralized and expensive healthcare system (45).

The problem is particularly severe when treatment requires frequent hospital visits over many months. A family traveling hundreds of kilometers for chemotherapy, laboratory investigations, transfusion support, or review may face cumulative transportation and accommodation costs that exceed their financial capacity. For AYAs, additional barriers may include interruption of education, employment responsibilities, concerns about fertility, psychosocial distress, and increasing independence from parents or caregivers (46). Treatment abandonment should therefore be understood as a health-system failure as much as a patient-level challenge. A cancer program that successfully initiates therapy but cannot retain patients throughout treatment has not achieved effective access to care. The solution requires proactive rather than reactive intervention. Patient navigation can identify families at risk of discontinuation before treatment is interrupted. Financial and transport assistance, nutritional support, temporary accommodation near treatment centers, flexible appointment scheduling, psychosocial counseling, telephone follow-up, and community-based monitoring can reduce practical barriers. Clear communication about treatment duration, expected adverse effects, warning signs, and the consequences of interruption can strengthen family engagement.

### The AYA oncology gap

AYAs with cancer occupy a vulnerable intersection between pediatric and adult oncology, creating a distinct gap in diagnosis, treatment, research, psychosocial support, and survivorship. Although AYAs develop cancers with unique biological and clinical characteristics, many health systems lack dedicated pathways that recognize their developmental and social needs. Consequently, young people may experience delayed diagnosis, inappropriate referral, fragmented treatment, and inadequate transition between pediatric and adult services (47). The diagnostic challenge is particularly important because cancer is often perceived as an uncommon disease in young people. Persistent fatigue, unexplained weight loss, bone pain, headaches, lymphadenopathy, abdominal masses, or other conerning symptoms may initially be attributed to infection, stress, injury, or benign conditions. Delayed recognition can result in more advanced disease at diagnosis and may reduce opportunities for less intensive, more effective treatment (48). Once diagnosed, AYAs may encounter additional barriers to appropriate therapy. Clinical trials and treatment protocols are frequently divided into pediatric and adult structures, leaving some patients inadequately represented in either. This can limit access to age-appropriate precision therapies and evidence-based clinical research. The biological heterogeneity of AYA cancers further emphasizes the need for molecular characterization and risk-adapted treatment.

The AYA cancer experience also extends beyond tumor control. Adolescence and young adulthood are critical periods for education, employment, relationships, sexual development, fertility, identity formation, and increasing independence. Cancer and its treatment can disrupt each of these domains. Fertility preservation, reproductive counseling, mental-health support, educational continuity, employment assistance, and age-appropriate communication should therefore be integrated into routine oncology care rather than treated as optional services (49). Transition between pediatric and adult oncology is another major vulnerability. Survivors may become lost during transfer if there is no structured transition plan, resulting in gaps in surveillance and management of late effects. A coordinated transition pathway should provide a comprehensive treatment summary, individualized survivorship plan, clear responsibility for follow-up, and active engagement of the young person in healthcare decisions (50). The AYA oncology gap is particularly pronounced in resource-constrained settings, where specialist services are centralized and survivorship programs may be limited. Digital health, tele-oncology, multidisciplinary networks, community-based follow-up, and regional referral systems can help extend specialist expertise beyond major cancer centers.

### Survivorship: from cure to lifelong health

Surviving cancer is a major therapeutic achievement, but for children, AYAs, survival should not represent the end of oncology care. Because many patients diagnosed during childhood or young adulthood may live for decades after treatment, the consequences of cancer and its therapy can extend far beyond remission. Survivorship must therefore evolve from a passive period of follow-up into an active, individualized strategy for preserving lifelong health, function, and quality of life (49). Cancer therapies can produce late effects involving the cardiovascular, endocrine, neurological, renal, pulmonary, reproductive, and musculoskeletal systems. Survivors may also experience neurocognitive difficulties, chronic fatigue, psychological distress, impaired fertility, secondary malignancies, educational disruption, and social or employment challenges. The magnitude of these risks depends on the cancer itself, age at treatment, cumulative drug exposure, radiotherapy fields and doses, surgery, complications, and individual susceptibility (51). This makes survivorship another essential dimension of precision oncology. Every survivor should ideally have an individualized treatment history documenting diagnosis, chemotherapy exposures, radiotherapy, surgery, immunotherapy, cellular therapy, major complications, and other relevant interventions. This information can function as a long-term risk map, allowing surveillance to be tailored according to previous treatment exposures rather than applying identical follow-up to every survivor. For example, survivors exposed to potentially cardiotoxic therapies may require long-term cardiovascular assessment, while those receiving therapies affecting endocrine organs may require endocrine and growth monitoring. Patients exposed to gonadotoxic treatment may require fertility and reproductive evaluation. Those with previous central nervous system disease or treatment may need neurocognitive and educational assessment. Survivorship must also address the psychosocial dimensions of recovery. Returning to school, securing employment, developing relationships, managing body image, establishing independence, maintaining mental health, and achieving reproductive goals can be as important to a young survivor as remaining free of cancer. AYA survivors may require additional support during the transition from intensive treatment to independent adult healthcare.

### Fertility and reproductive health

Cancer treatment during childhood and adolescence can affect gonadal function and future fertility. Because reproductive consequences may not become clinically apparent until adulthood, fertility preservation should be considered early. Approaches may include sperm or oocyte/embryo preservation where appropriate, ovarian tissue cryopreservation, testicular tissue preservation in selected circumstances, and fertility counseling. In many LMICs, however, fertility preservation services are limited or unaffordable. An equity-oriented model should therefore distinguish between interventions that require highly specialized technology and interventions that can be implemented universally, such as early counseling, documentation of gonadotoxic exposure, referral pathways, and reproductive surveillance (52).

### Psychosocial and neurocognitive survivorship

The psychosocial consequences of cancer can be substantial. Children may experience interrupted schooling, social isolation, altered body image, developmental difficulties, and anxiety. AYAs may face disruption of higher education, employment, relationships, reproductive plans, and financial independence. Neurocognitive effects may occur following central nervous system disease or certain treatment exposures. Survivorship programs should therefore incorporate educational assessment and rehabilitation when necessary. Mental health services should not be treated as optional additions. They should form part of integrated oncology care. The goal is to move from survival with disability toward survival with restored function and participation (53).

### Health equity and social determinants of cancer outcomes

Biological precision cannot overcome structural inequality. Cancer outcomes are influenced by socioeconomic status, geographic location, education, health literacy, transportation, nutrition, housing, employment, insurance coverage, and social support. A child living far from an oncology center may experience treatment interruption because travel is unaffordable. A caregiver may miss appointments because attending treatment means losing income. A young adult may discontinue therapy because of employment obligations. These factors should be documented as clinical variables rather than treated as irrelevant background information. A modern oncology record should therefore capture selected social determinants that directly influence treatment feasibility and outcomes. This creates the possibility of socially informed precision oncology (54).

### Selected evidence from LMIC settings: illustrating the equity gap in pediatric and AYA cancer care

The challenges of pediatric and AYA cancer care in LMICs are best understood through selected examples that demonstrate how inequity can arise at different points along the cancer-care continuum. These examples are not intended to provide an exhaustive country-by-country comparison or to suggest that all LMICs experience identical barriers. Rather, they illustrate recurring patterns involving delayed diagnosis, fragmented referral systems, treatment abandonment, limited diagnostic and therapeutic capacity, and unequal survival. Importantly, the magnitude and determinants of these problems vary according to national health-system organization, cancer infrastructure, socioeconomic conditions, disease spectrum, and availability of pediatric oncology services (55).

#### Diagnostic delay and late presentation

Delayed diagnosis remains an important barrier to successful childhood cancer treatment in many LMIC settings. Pediatric cancers often present with nonspecific symptoms that overlap with common infectious, nutritional, and hematological conditions. Where primary healthcare providers have limited exposure to childhood malignancies and referral pathways are poorly defined, children may undergo repeated consultations or empirical treatment before cancer is suspected. Geographic distance from specialist facilities, transportation costs, diagnostic fees, and limited access to pathology and imaging can further extend the interval between symptom onset and definitive diagnosis. Evidence from sub-Saharan Africa illustrates the consequences of these limitations. Studies from countries including Nigeria, Kenya, Uganda, Tanzania, and Ghana have described late presentation and delays across different stages of the diagnostic pathway. These delays should not be attributed solely to families; they frequently reflect a combination of caregiver awareness, primary-care recognition, referral efficiency, diagnostic availability, and socioeconomic barriers. In some settings, children reach specialist oncology services only after substantial disease progression, reducing the therapeutic window for potentially curative treatment (56, 57).

Nigeria provides an illustrative example of the interaction between disease burden and health-system constraints. Pediatric oncology services are concentrated in relatively few tertiary centers, while many patients initially present to primary or secondary facilities where diagnostic resources and pediatric oncology expertise may be limited. Consequently, the pathway from symptom recognition to definitive diagnosis can involve multiple healthcare encounters. Similar challenges have been described in other African settings, although the magnitude differs between countries and institutions. These observations support a fundamental principle of precision oncology: diagnostic precision begins before molecular testing. Strengthening recognition of cancer warning signs, establishing clear referral criteria, improving laboratory and imaging access, and developing efficient specimen-transfer systems may produce substantial gains even where comprehensive genomic profiling is unavailable (58).

#### Referral barriers and geographic inequity

Specialist pediatric oncology services in many LMICs are geographically concentrated. This creates a structural disparity between patients living near major cancer centers and those living in rural or underserved regions. Families may need to travel long distances, relocate temporarily, or repeatedly return to tertiary hospitals for diagnostic procedures, chemotherapy, transfusion support, imaging, or treatment monitoring. The referral pathway can therefore become a determinant of outcome in its own right. A child with a potentially curable malignancy may experience several delays: recognition at the community level, referral from primary to secondary care, referral to a tertiary center, pathology confirmation, initiation of treatment, and subsequent return for monitoring. In countries with large geographic areas and uneven specialist distribution, decentralization and networking may provide a practical solution. Rather than requiring every facility to develop comprehensive pediatric oncology capability, regional systems can connect primary and secondary facilities with designated pediatric oncology centers through standardized referral protocols, teleconsultation, pathology networks, and specimen transportation. Such approaches can shorten referral intervals while retaining specialist oversight. For AYA patients, referral fragmentation may be compounded by the division between pediatric and adult oncology services. Adolescents may not fit easily within either pathway, potentially resulting in delayed referral, inconsistent treatment protocols, or loss of continuity during transition (59, 60).

#### Treatment abandonment

Treatment abandonment represents another major manifestation of inequity in childhood cancer care. It is particularly important in settings where families bear substantial direct and indirect costs of treatment. Even when chemotherapy or other cancer therapies are nominally available, transportation, accommodation, food, caregiver income loss, medication costs, and repeated hospital visits may create financial pressures that make treatment completion difficult. Studies from several LMIC settings have reported treatment abandonment as an important contributor to poor childhood cancer outcomes. The frequency varies substantially between countries, institutions, cancer types, treatment protocols, and definitions used in individual studies. Consequently, reported rates should not be interpreted as a single representative estimate for all LMICs. Treatment abandonment should be understood within its social and health-system context. Families may discontinue treatment because they cannot afford transportation, live far from the treatment center, experience severe treatment toxicity, lack accommodation, misunderstand the expected duration of therapy, or encounter medicine and service interruptions. These factors indicate that abandonment is not simply a measure of individual adherence; it can also be a marker of health-system accessibility and social protection. Precision oncology can respond to this problem by incorporating social risk into clinical care. Treatment-navigation programs, transportation assistance, accommodation support, caregiver counseling, appointment reminders, decentralized supportive services, and early identification of families at risk of treatment interruption can complement biologically precise treatment (61, 62).

#### Health-system capacity and diagnostic infrastructure

The effectiveness of precision oncology depends on the diagnostic infrastructure supporting it. In several LMIC settings, limitations may occur at multiple levels, including pathology, immunohistochemistry, flow cytometry, cytogenetics, molecular diagnostics, radiology, blood transfusion, microbiology, and laboratory quality assurance. For hematological malignancies, inadequate access to flow cytometry or molecular testing may limit accurate classification and risk stratification. For solid tumors, limited histopathology, immunohistochemistry, advanced imaging, and molecular testing can complicate integrated diagnosis. Specimen transportation is another important consideration because tissue or blood samples may need to travel from regional hospitals to centralized reference laboratories. These limitations have direct implications for precision oncology. A sequencing platform alone cannot create a precision oncology system if tissue procurement is unreliable, pathology interpretation is inconsistent, or patients cannot access the treatment indicated by the molecular result. Consequently, strengthening basic diagnostic services should be regarded as a component of precision oncology rather than a preliminary step unrelated to it. A tiered model may be particularly appropriate. Essential pathology and laboratory services can form the foundation, followed by regional access to immunophenotyping and targeted molecular tests, with comprehensive genomic profiling concentrated in appropriately equipped reference centers. Such networks can allow advanced diagnostics to be shared across multiple institutions rather than duplicated at every facility (63, 64).

#### Treatment capacity and supportive-care constraints

Treatment disparities extend beyond access to anticancer drugs. Pediatric oncology requires reliable supportive-care infrastructure, including blood products, antimicrobial therapy, nutritional support, pain management, management of treatment-related complications, intensive care when necessary, and appropriately trained healthcare personnel. In resource-constrained settings, interruptions in chemotherapy may occur because of medicine shortages, equipment failure, staffing limitations, financial barriers, or lack of supportive-care capacity. Treatment-related infections and other complications may become particularly consequential when microbiology, intensive-care, transfusion, or antimicrobial resources are limited. This reinforces the concept that therapeutic precision must incorporate supportive-care precision. Selecting the correct chemotherapy or targeted therapy is insufficient if the healthcare system cannot safely deliver the treatment or manage predictable toxicities (65, 66).

#### Disparities in pediatric cancer outcomes

The cumulative effect of diagnostic, referral, treatment, and supportive-care barriers is reflected in the marked international variation in childhood cancer survival. Children treated in high-income settings have achieved high survival for many common pediatric malignancies, whereas survival remains substantially lower in many LMICs. International estimates have consistently identified a large global childhood cancer survival gap, with survival differences reflecting multiple interacting factors rather than a single cause. The disparity is particularly evident for malignancies that are highly curable when diagnosed and treated appropriately. Acute lymphoblastic leukemia, Hodgkin lymphoma, Wilms tumor, retinoblastoma, and several other childhood cancers can achieve excellent outcomes under appropriate treatment conditions. When survival is substantially poorer in resource-constrained environments, the difference highlights potentially modifiable failures in diagnosis, treatment delivery, supportive care, and continuity. This distinction is important for the precision oncology agenda. Precision medicine should not focus exclusively on rare molecular targets while patients with common, highly curable cancers continue to experience preventable diagnostic and treatment barriers. Equitable precision oncology must therefore combine advanced biological stratification with reliable delivery of established curative therapies (54, 67).

#### AYA cancer care in LMICs

The evidence base for AYA cancer in LMICs is less developed than that for childhood cancer, partly because adolescents and young adults are often distributed across pediatric and adult oncology systems. Cancer registration may also be inconsistent across this age range, making the burden and outcomes of AYA malignancies difficult to characterize accurately. AYA patients may experience barriers related to delayed recognition, limited age-specific oncology services, lack of clinical trial participation, fertility preservation, mental-health services, education, employment, and transition between pediatric and adult care. These challenges are particularly important in settings where cancer services are already centralized. An equity-focused AYA precision oncology model should therefore incorporate age-appropriate communication, reproductive and fertility counseling, psychosocial support, education and employment considerations, and structured transition pathways. These services should not be regarded as optional additions to molecular oncology; they are integral components of patient-centered precision care (68).

#### Strategic lessons for precision oncology in LMICs

The evidence from LMIC settings supports several practical conclusions. First, earlier recognition and efficient referral are precision interventions because accurate molecular characterization has little value when diagnosis occurs too late. Second, pathology and laboratory strengthening are foundational investments that enable subsequent genomic and molecular precision. Third, treatment abandonment requires health-system and social interventions, not simply greater emphasis on adherence. Fourth, supportive care is an essential component of therapeutic precision. Finally, survivorship must be incorporated from diagnosis, particularly as increasing numbers of children and AYA patients survive cancer. These lessons support a staged and networked model rather than an approach that attempts to reproduce high-income-country oncology infrastructure immediately. Regional diagnostic laboratories, telepathology, shared molecular platforms, standardized referral pathways, treatment-navigation programs, essential-medicine systems, and longitudinal cancer registries can provide intermediate solutions while advanced genomic technologies are progressively introduced (69, 70).

#### Integrating the evidence into the proposed roadmap

Taken together, selected evidence from Nigeria, Kenya, and other LMIC settings demonstrates that inequity occurs across multiple interconnected levels rather than at a single point in the cancer pathway. Diagnostic delay can lead to advanced disease; centralized specialist services can amplify geographic barriers; financial and logistical pressures can contribute to treatment abandonment; inadequate pathology can restrict molecular classification; and insufficient survivorship infrastructure can leave long-term complications unrecognized (71, 72).

The proposed precision oncology roadmap therefore places equity across the entire continuum:

Recognition → Referral → Accurate diagnosis → Molecular characterization → Risk stratification → Accessible treatment → Supportive care → Response monitoring → Survivorship → Lifelong follow-up.

This framework also emphasizes that implementation should be adapted to local epidemiology, infrastructure, workforce, financing, and health-system organization. The objective is not to impose a single model on all LMICs, but to establish a scalable pathway through which each health system can progressively improve the precision, accessibility, continuity, and equity of pediatric and AYA cancer care (73, 74).

## Conclusion

Achieving equity in pediatric and AYA cancer care requires precision across the entire cancer continuum, from early recognition and accurate diagnosis to risk-adapted treatment and lifelong survivorship. Precision oncology should therefore extend beyond genomic profiling to integrate clinical characteristics, tumor biology, treatment response, toxicity risk, socioeconomic circumstances, and health-system constraints. Earlier recognition, robust diagnostic capacity, dynamic risk stratification, individualized supportive care, treatment adherence, and coordinated survivorship services are essential components of this broader model. In resource-constrained settings, equitable precision oncology should combine scalable foundational services with progressively accessible molecular and digital technologies rather than relying exclusively on high-cost genomic approaches. Strengthening referral networks, pathology and laboratory capacity, essential medicines, multidisciplinary care, data systems, and survivorship infrastructure can substantially reduce preventable losses across the cancer pathway. Ultimately, the goal is not simply to identify the right therapy for the right tumor, but to ensure that every child and AYA has timely access to the right diagnosis, appropriate treatment, continuous support, and lifelong follow-up. A whole-pathway precision framework can therefore transform pediatric and AYA oncology from a model focused primarily on survival into one centered on equitable cure, functional recovery, quality of life, and lifelong health.

## References

[1] Filbin M Monje M . Developmental origins and emerging therapeutic opportunities for childhood cancer. Nat Med. (2019) 25:367–76. doi: 10.1038/s41591-019-0383-9 30842674 PMC6631320

[2] Zahnreich S Schmidberger H . Childhood cancer: Occurrence, treatment and risk of second primary malignancies. Cancers. (2021) 13:2607. doi: 10.3390/cancers13112607 34073340 PMC8198981

[3] Srikanthan A . Precision medicine for adolescent and young adult (AYA) oncology. J Pers Med. (2023) 13:1449. doi: 10.3390/jpm13101449 37888060 PMC10608298

[4] Molla G Bitew M . The future of cancer diagnosis and treatment: Unlocking the power of biomarkers and personalized molecular-targeted therapies. J Mol Pathol. (2025) 6:20. doi: 10.3390/jmp603002030654563

[5] Ricci AM Emeny RT Bagley PJ Blunt HB Butow ME Morgan A . Causes of childhood cancer: A review of the recent literature: Part I-childhood factors. Cancers (Basel). (2024) 16:1297. doi: 10.3390/cancers16071297 42073643 PMC13114923

[6] Dutta R Vallurupalli M McVeigh Q Huang FW Rebbeck TR . Understanding inequities in precision oncology diagnostics. Nat Cancer. (2023) 4:787–94. doi: 10.1038/s43018-023-00568-1 37248397

[7] Nair N Schlumbrecht M . Existing health inequities in the treatment of advanced and metastatic cancers. Curr Oncol Rep. (2024) 26:1553–62. doi: 10.1007/s11912-024-01617-3 39495424 PMC11646267

[8] Lustberg MB Kuderer NM Desai A Bergerot C Lyman GH . Mitigating long-term and delayed adverse events associated with cancer treatment: Implications for survivorship. Nat Rev Clin Oncol. (2023) 20:527–42. doi: 10.1038/s41571-023-00776-9 37231127 PMC10211308

[9] Butnari V Scantlebury P Green T Pattni D Mansuri A Chinnery W . The crucial role of early diagnosis for patients and the nation, understanding the costs of late-stage cancer diagnosis from a large district general hospital in England. Cost Eff Resour Alloc. (2025) 23:60. doi: 10.1186/s12962-025-00657-1 41152849 PMC12570495

[10] Ang SP Lee E Chia JE Iglesias M Di Vanna M Shambhavi S . Time-to-treatment initiation and its effect on all-cause mortality: Insights from the surveillance, epidemiology, and end results database. World J Oncol. (2025) 16:286–94. doi: 10.14740/wjon2584 40556964 PMC12185122

[11] Mikkelsen MK Pham LTD Sotelo C Kelley M Edwards M Lion RR . The global clinical trial landscape for children and adolescents with cancer. JAMA Netw Open. (2026) 9:e2552510. doi: 10.1001/jamanetworkopen.2025.52510 41493780 PMC12776201

[12] Santero M Ortiz Sequeira R Cruz Paixao M Muñoz Martinez M Mazorra Roig P Chantada G . Childhood cancer survival in low- and middle-income countries and the global south: Emerging evidence and critical gaps from a scoping review of observational studies. EJC Paediatr Oncol. (2025) 6:None. doi: 10.1016/j.ejcped.2025.100422 41368027 PMC12682699

[13] Brlek P Škaro V Hrvatin N Bulić L Petrović A Projić P . Advances in precision oncology: From molecular profiling to regulatory-approved targeted therapies. Cancers (Basel). (2025) 17:3500. doi: 10.3390/cancers17213500 41228293 PMC12607717

[14] Qiao D Wang RC Wang Z . Precision oncology: Current landscape, emerging trends, challenges, and future perspectives. Cells. (2025) 14:1804. doi: 10.3390/cells14221804 41294857 PMC12651332

[15] Beauchemin MP Roth ME Parsons SK . Reducing adolescent and young adult cancer outcome disparities through optimized care delivery: A blueprint from the children's oncology group. J Adolesc Young Adult Oncol. (2023) 12:314–23. doi: 10.1089/jayao.2022.0136 36716260 PMC10282820

[16] Fragkandrea I Nixon JA Panagopoulou P . Signs and symptoms of childhood cancer: A guide for early recognition. Am Fam Physician. (2013) 88:185–92. 23939697

[17] Matolengwe A Murray D Okafor UB . The challenges of implementing a health referral system in South Africa: A qualitative study. Risk Manag Healthc Policy. (2024) 17:855–64. doi: 10.2147/RMHP.S450998 38623578 PMC11017115

[18] Uribe-Ortiz LV Garza-Ornelas BM Vázquez-Fernández AC Castorena-Torres F Rodríguez-De-Ita J . Exploring knowledge of parents and caregivers on cancer symptoms in children: An observational study regarding the need for educational tools and health promotion in low- and middle-income countries. BMC Pediatr. (2022) 22:638. doi: 10.1186/s12887-022-03686-4 36333691 PMC9635160

[19] Soares Martins QC Gomes de Morais Fernandes FC Pereira Santos VE Guerra Azevedo I Góes de Carvalho Nascimento LS Dos Santos Xavier CC . Factors associated with the detection of childhood and adolescent cancer in primary health care: A prospective cross-sectional study. J Multidiscip Healthc. (2020) 13:329–37. doi: 10.2147/JMDH.S225641 32280234 PMC7125305

[20] Tefferi A Hanson CA Inwards DJ . How to interpret and pursue an abnormal complete blood cell count in adults. Mayo Clin Proc. (2005) 80:923–36. doi: 10.4065/80.7.923 16007898 PMC7127472

[21] Caraiani C Yi D Petresc B Dietrich C . Indications for abdominal imaging: When and what to choose? J Ultrason. (2020) 20:e43–54. doi: 10.15557/JoU.2020.0008 32320166 PMC7266076

[22] White C Robinson S Macleod U Kelly C . GP referral to rapid diagnostic centres for non-specific cancer symptoms: A qualitative study. BJGP Open. (2025) 9:BJGPO.2024.0179. doi: 10.3399/BJGPO.2024.0179 40015746 PMC12728846

[23] Alnasser Y Proaño A Loock C Chuo J Gilman RH . Telemedicine and pediatric care in rural and remote areas of middle-and-low-income countries: Narrative review. J Epidemiol Glob Health. (2024) 14:779–86. doi: 10.1007/s44197-024-00214-8 38478166 PMC11442723

[24] Phillips CR Haase JE Broome ME Carpenter JS Frankel RM . Connecting with healthcare providers at diagnosis: Adolescent/young adult cancer survivors' perspectives. Int J Qual Stud Health Well-being. (2017) 12:1325699. doi: 10.1080/17482631.2017.1325699 28617094 PMC5510205

[25] Kuhlman KR Delacruz P Lopez S Tran MM Rodgers EL . Barriers to mental health services among at-risk and symptomatic adolescents in the community. Child Adolesc Psychiatry Ment Health. (2025) 19:121. doi: 10.1186/s13034-025-00978-2 41194107 PMC12590828

[26] Natale-Pereira A Enard KR Nevarez L Jones LA . The role of patient navigators in eliminating health disparities. Cancer. (2011) 117:3543–52. doi: 10.1002/cncr.26264 21780089 PMC4121958

[27] Fahim YA Hasani IW Kabba S Ragab WM . Artificial intelligence in healthcare and medicine: Clinical applications, therapeutic advances, and future perspectives. Eur J Med Res. (2025) 30:848. doi: 10.1186/s40001-025-03196-w 40988064 PMC12455834

[28] Obeagu EI Ogenyi FC . Artificial intelligence in African malaria control programs: Opportunities and risks. Ann Med Surg (Lond). (2025) 87:8664–70. doi: 10.1097/MS9.0000000000004259 41377369 PMC12688759

[29] Thenrajan T Alwarappan S Wilson J . Molecular diagnosis and cancer prognosis-a concise review. Diagnostics (Basel). (2023) 13:766. doi: 10.3390/diagnostics13040766 36832253 PMC9955694

[30] Hassan M Shahzadi S Kloczkowski A . Harnessing artificial intelligence in pediatric oncology diagnosis and treatment: A review. Cancers (Basel). (2025) 17:1828. doi: 10.3390/cancers17111828 40507308 PMC12153614

[31] Singh SB Sarrami AH Gatidis S Varniab ZS Chaudhari A Daldrup-Link HE . Applications of artificial intelligence for pediatric cancer imaging. AJR Am J Roentgenol. (2024) 223:e2431076. doi: 10.2214/AJR.24.31076 38809123 PMC11874589

[32] Udrycka K Rutkowski K Osnytskyy A Małecka-Wojciesko E . Tumor characteristics and clinical features of the patient as prognostic factors in PDAC. Cancers (Basel). (2025) 17:3350. doi: 10.3390/cancers17203350 41154404 PMC12562580

[33] Kania E Janica M Nesterowicz M Modzelewski W Cybulski M Janica J . Advances and challenges in prostate cancer diagnosis: A comprehensive review. Cancers. (2025) 17:2137. doi: 10.3390/cancers17132137 40647437 PMC12248846

[34] Daw S Hasenclever D Mascarin M Fernández-Teijeiro A Balwierz W Beishuizen A . Risk and response adapted treatment guidelines for managing first relapsed and refractory classical Hodgkin lymphoma in children and young people. Recommendations from the EuroNet Pediatric Hodgkin Lymphoma Group. Hemasphere. (2020) 4:e329. doi: 10.1097/HS9.0000000000000329 32072145 PMC7000476

[35] Zafar S Hafeez A Shah H Mutiullah I Ali A Khan K . Emerging biomarkers for early cancer detection and diagnosis: Challenges, innovations, and clinical perspectives. Eur J Med Res. (2025) 30:760. doi: 10.1186/s40001-025-03003-6 40826140 PMC12359849

[36] Ahmed HM Al-Hussainy AF Mustafa WW Jyothi SR Nayak PP Janney JB . Circulating tumor DNA dynamics predict pathological response and guide therapy personalization in the neoadjuvant setting. Discov Oncol. (2026) 17:365. doi: 10.1007/s12672-026-04495-2 41611911 PMC12936245

[37] Obeagu EI . Transcriptomic and epigenomic insights into ovarian cancer: A bioinformatics perspective - a narrative review. Ann Med Surg (Lond). (2026) 88:5073–9. doi: 10.1097/MS9.0000000000004798 42583634 PMC13461056

[38] Ferrero JM Bouriga R Gal J Milano G . De-escalating anticancer treatment: Watch your step. Cancers (Basel). (2025) 17:2474. doi: 10.3390/cancers17152474 40805173 PMC12346573

[39] Barnett M McDonnell G DeRosa A Schuler T Philip E Peterson L . Psychosocial outcomes and interventions among cancer survivors diagnosed during adolescence and young adulthood (AYA): A systematic review. J Cancer Surviv. (2016) 10:814–31. doi: 10.1007/s11764-016-0527-6 26920873 PMC5001943

[40] Lauschke VM Ingelman-Sundberg M . The evolving landscape of pharmacogenomics: Current achievements and future directions. Pharmacol Rev. (2026) 78:100122. doi: 10.1016/j.pharmr.2026.100122 41806469 PMC13084611

[41] Smith MA Reaman GH . Remaining challenges in childhood cancer and newer targeted therapeutics. Pediatr Clin North Am. (2015) 62:301–12. doi: 10.1016/j.pcl.2014.09.018 25435124 PMC4336187

[42] Molla YM Shad A Wali R Yimer MA . The magnitude and associated factors of childhood cancer treatment abandonment at the university of Gondar comprehensive specialized hospital, Ethiopia. Sci Rep. (2025) 15:10534. doi: 10.1038/s41598-025-90493-3 40148396 PMC11950395

[43] Back AL Young JP McCown E Engelberg RA Vig EK Reinke LF . Abandonment at the end of life from patient, caregiver, nurse, and physician perspectives: Loss of continuity and lack of closure. Arch Intern Med. (2009) 169:474–9. doi: 10.1001/archinternmed.2008.583 19273777 PMC2918275

[44] Thomson MD Van Houtven CH Xu R Siminoff LA . The many "costs" of transportation: Examining what cancer caregivers experience as transportation obstacles. Cancer Med. (2023) 12:17356–64. doi: 10.1002/cam4.6351 37485648 PMC10501274

[45] Ramphal R Aubin S Czaykowski P De Pauw S Johnson A McKillop S . Adolescent and young adult cancer: Principles of care. Curr Oncol. (2016) 23:204–9. doi: 10.3747/co.23.3013 27330350 PMC4900833

[46] Scheel BI Holtedahl K . Symptoms, signs, and tests: The general practitioner's comprehensive approach towards a cancer diagnosis. Scand J Prim Health Care. (2015) 33:170–7. doi: 10.3109/02813432.2015.1067512 26375323 PMC4750720

[47] Reinman L Coons HL Sopfe J Casey R . Psychosexual care of adolescent and young adult (AYA) cancer survivors. Children (Basel). (2021) 8:1058. doi: 10.3390/children8111058 34828771 PMC8618923

[48] Strini V Daicampi C Trevisan N Marinetto A Prendin A Marinelli E . Transition of care in pediatric oncohematology: A systematic literature review. Acta BioMed. (2020) 91:48–64. doi: 10.23750/abm.v91i6-S.9876 32573506 PMC7975840

[49] Adams SC Herman J Lega IC Mitchell L Hodgson D Edelstein K . Young adult cancer survivorship: Recommendations for patient follow-up, exercise therapy, and research. JNCI Cancer Spectr. (2020) 5:pkaa099. doi: 10.1093/jncics/pkaa099 33681702 PMC7919337

[50] Harrington J White J . The late medical effects of cancer treatments: a growing challenge for all medical professionals. Clin Med (Lond). (2017) 17:137–9. doi: 10.7861/clinmedicine.17-2-137 28365623 PMC6297612

[51] Korenblum C Taylor RM Fern LA Hough R Wickramasinghe B . Factors affecting psychosocial distress in adolescents and young adults with cancer: BRIGHTLIGHT cross-sectional and longitudinal cohort study results. Cancers (Basel). (2025) 17:1196. doi: 10.3390/cancers17071196 40227784 PMC11987992

[52] Chin ZS Ghodrati A Foulger M Demirkhanyan L Gondi CS . Beyond barriers: Achieving true equity in cancer care. Curr Oncol. (2025) 32:349. doi: 10.3390/curroncol32060349 40558292 PMC12191551

[53] Ajagbe S . Contemporary challenges in cancer care in LMICS: Perspectives from awareness, prevention, screening, and health financing research. Ann Ib Postgrad Med. (2026) 21:6–7. PMC13399215 42499468

[54] Gizaw AT Woldie I Yilma AN Tadesse M Sisay AL Sebero FM . Barriers to timely diagnosis and treatment of childhood cancer in Sub-Sahara Africa: a qualitative systematic review. BMC Cancer. (2025) 25:1600. doi: 10.1186/s12885-025-15092-x 41107757 PMC12534927

[55] Ain RU Rahman AA Tariq A Faizan M . Delay in diagnosis and treatment of childhood cancers in a developing country: A cross-sectional patient-based survey at country's largest pediatric oncology center. Pak J Med Sci. (2025) 41:1267–73. doi: 10.12669/pjms.41.5.9663 40469167 PMC12130928

[56] Chukwu OA Adewole IF Denburg A Essue BM . Improving childhood cancer medicines access in developing countries: Towards an implementation framework to inform the Global Platform for Access to Childhood Cancer Medicines for Nigeria. PloS Glob Public Health. (2024) 4:e0003275. doi: 10.1371/journal.pgph.0003275 39240943 PMC11379258

[57] Chiu M Ali A Leung F Dong C Pechlivanoglou P Hodgson D . Sociodemographic factors and utilization of pediatric oncology satellite clinics in Ontario, Canada. JAMA Netw Open. (2024) 7:e2452063. doi: 10.1001/jamanetworkopen.2024.52063 39724373 PMC11672161

[58] Walling EB Fiala M Connolly A Drevenak A Gehlert S . Challenges associated with living remotely from a pediatric cancer center: A qualitative study. J Oncol Pract. (2019) 15:e219-e229. doi: 10.1200/JOP.18.00115 30702962 PMC6416804

[59] Mirutse MK Tolla MT Memirie ST Palm MT Hailu D Abdi KA . The magnitude and perceived reasons for childhood cancer treatment abandonment in Ethiopia: from health care providers' perspective. BMC Health Serv Res. (2022) 22:1014. doi: 10.1186/s12913-022-08188-8 35941600 PMC9361525

[60] Hazarika M Mishra R Saikia BJ Bhuyan C Nyuthe CW Sarma A . Causes of treatment abandonment of pediatric cancer patients – experience in a regional cancer centre in North East India. Asian Pac J Cancer Prev. (2019) 20:1133–7. doi: 10.31557/APJCP.2019.20.4.1133 31030486 PMC6948912

[61] Ahmad Z Rahim S Zubair M Abdul-Ghafar J . The new era of precision oncology. Practicing pathology in low resource countries and settings. Int J Gen Med. (2026) 19:593156. doi: 10.2147/IJGM.S593156 41913905 PMC13033194

[62] Mandefro B Berta DM Kelem A Teketelew BB Melkamu A Much Y . The role of advanced diagnostics on precision medicine in hemato oncology. Discov Oncol. (2025) 16:1525. doi: 10.1007/s12672-025-03169-9 40788517 PMC12339839

[63] Freedman JL Beeler DM Bowers A Bradford N Cheung YT Davies M . Supportive care in pediatric oncology: Opportunities and future directions. Cancers (Basel). (2023) 15:5549. doi: 10.3390/cancers15235549 38067252 PMC10705083

[64] Obeagu EI Ezeanya CU Ogenyi FC Ifu DD . Big data analytics and machine learning in hematology: Transformative insights, applications and challenges. Med (Baltimore). (2025) 104:e41766. doi: 10.1097/MD.0000000000041766 40068020 PMC11902945

[65] Aristizabal P Winestone LE Umaretiya P Bona K . Disparities in pediatric oncology: The 21st century opportunity to improve outcomes for children and adolescents with cancer. Am Soc Clin Oncol Educ Book. (2021) 41:e315-e326. doi: 10.1200/EDBK_320499 34061564 PMC9126642

[66] Tonorezos ES Cohn RJ Glaser AW Lewin J Poon E Wakefield CE . Long-term care for people treated for cancer during childhood and adolescence. Lancet. (2022) 399:1561–72. doi: 10.1016/S0140-6736(22)00460-3 35430023 PMC9082556

[67] Reuter K Freyer DR Milam J Mobley EM . Inconsistent reporting of adolescent and young adult (AYA) status in non-interventional cancer research: a critical barrier to evidence usability. BMJ Open. (2026) 16:e107742. doi: 10.1136/bmjopen-2025-107742 42526924 PMC13422801

[68] Radich JP Briercheck E Chiu DT Menon MP Sala Torra O Yeung CCS . Precision medicine in low- and middle-income countries. Annu Rev Pathol. (2022) 17:387–402. doi: 10.1146/annurev-pathol-042320-034052 35073168 PMC9275191

[69] Obeagu EI . Revolutionizing hematological disorder diagnosis: unraveling the role of artificial intelligence. Ann Med Surg (Lond). (2025) 87:3445–57. doi: 10.1097/MS9.0000000000003227 40486570 PMC12140674

[70] Nkengasong JN Yao K Onyebujoh P . Laboratory medicine in low-income and middle-income countries: progress and challenges. Lancet. (2018) 391:1873–5. doi: 10.1016/S0140-6736(18)30308-8 29550031 PMC5962422

[71] Mohapatra D Meena JP Gupta AK Seth R . Evolution of therapy in pediatric ALL from aminopterin to chimeric antigen receptor T-cell therapy: Global advances, Indian adaptations, and lessons for low- and middle-income countries. JCO Glob Oncol. (2026) 12:e2500478. doi: 10.1200/GO-25-00478 41843833

[72] Mohapatra D Pushpam D Bakhshi S Sahoo RK Sharawat SK Agarwala S . Autologous stem cell transplantation for pediatric solid tumors in a resource-limited setting: a single-center experience of 15 years. Front Oncol. (2026) 16:1796956. doi: 10.3389/fonc.2026.1796956 42382384 PMC13314500

[73] Ferrari A Stark D Peccatori FA Fern L Laurence V Gaspar N . Adolescents and young adults (AYA) with cancer: A position paper from the AYA working group of the European Society for Medical Oncology (ESMO) and the European Society for Paediatric Oncology (SIOPE). ESMO Open. (2021) 6:100096. doi: 10.1016/j.esmoop.2021.100096 33926710 PMC8103533

[74] Yang EH Strohl HB Su HI . Fertility preservation before and after cancer treatment in children, adolescents, and young adults. Cancer. (2024) 130:344–55. doi: 10.1002/cncr.35108 37962199 PMC10841998

